# Radiomic Analysis and Liquid Biopsy in Preoperative CT of NSCLC: An Explorative Experience

**DOI:** 10.1111/1759-7714.70115

**Published:** 2025-07-03

**Authors:** Maria Paola Belfiore, Mario Sansone, Giovanni Ciani, Vittorio Patanè, Carlotta Genco, Roberta Grassi, Giovanni Savarese, Marco Montella, Riccardo Monti, Salvatore Cappabianca, Alfonso Reginelli

**Affiliations:** ^1^ Department of Precision Medicine University of Campania Luigi Vanvitelli Naples Italy; ^2^ Department of Electrical Engineering and Information Technology University of Naples “Federico II” Naples Italy; ^3^ AMES—Centro Polidiagnostico Strumentale, SRL Naples Italy

**Keywords:** ALK, genomics, nonsmall cell lung cancer, prognosis, radiomics, ROS1

## Abstract

**Background:**

Nonsmall cell lung cancer (NSCLC) remains a significant global health burden, necessitating advancements in diagnostic and prognostic strategies. Liquid biopsy and radiomics offer promising avenues for enhancing preoperative assessment and treatment planning in NSCLC.

**Methods:**

This prospective study enrolled 60 NSCLC patients who underwent both computed tomography (CT)‐guided biopsy and liquid biopsy. Radiomic features were extracted from CT images, and circulating tumor DNA (ctDNA) was sequenced to identify genetic mutations. Machine learning algorithms were employed to assess the association between radiomic features and gene mutations.

**Results:**

Among 57 patients with available data, associations between radiomic features and gene pairs mutation obtained from liquid biopsy exhibited moderate accuracy (approximately 0.60), with texture features demonstrating higher importance. However, when predicting the combined mutation status of gene pairs (e.g., EGFR and ROS1), the classification task involved three classes and yielded substantially lower accuracy (approximately 0.30), likely due to class imbalance and increased complexity.

**Discussion:**

Our findings demonstrate a moderate association between radiomic features and single gene mutations detected through liquid biopsy in NSCLC patients, with classification accuracies reaching approximately 0.60. In contrast, classification performance significantly declined (to ~0.30) when gene mutation pairs were used as targets, likely due to increased complexity and class imbalance. Notably, second‐order texture features showed the highest importance in the models. These preliminary results suggest that radiomics may capture aspects of tumor biology reflected in liquid biopsy, warranting further validation in larger, well‐balanced cohorts.

**Conclusion:**

The integration of liquid biopsy and radiomics holds promise for enhancing preoperative assessment and personalized treatment strategies in NSCLC. Further research on larger cohorts is warranted to validate the findings and translate them into clinical practice.

**Trial Registration:** University of Campania Trial Board UC20201112‐24997

## Graphical abstract

Radiomic features extracted from CT images were combined with ctDNA mutational data from liquid biopsy in NSCLC patients. Machine learning showed moderate accuracy (~0.60) for predicting single‐gene mutations, but lower performance (~0.30) for gene‐pair classifications. Texture‐based features were most informative. These findings highlight the potential of integrating radiomics and liquid biopsy for preoperative tumor profiling.

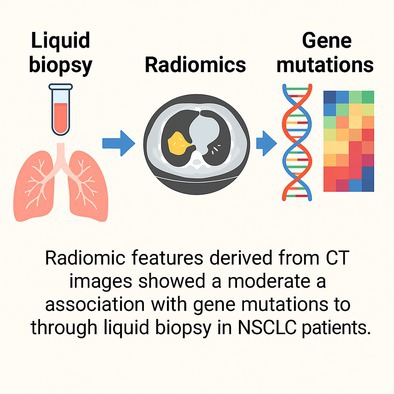

## 1 Introduction

Radiogenomics is an emerging field that aims to bridge the gap between imaging phenotypes and tumor genomics by associating quantitative radiomic features with specific molecular alterations [1, 2]. In the context of nonsmall cell lung cancer (NSCLC), a disease characterized by pronounced inter‐ and intratumoral heterogeneity and a wide spectrum of driver mutations, this integrative approach has the potential to refine diagnostic and prognostic tools and guide personalized treatment strategies [3, 4, 5, 6]. While conventional radiologic assessment plays a central role in NSCLC staging and treatment planning, it provides limited information about the [7, 8]. Conversely, tissue‐based genomic profiling offers insights into actionable mutations, yet suffers from spatial sampling bias, invasiveness, and limited feasibility for longitudinal monitoring [9, 10, 11]. To overcome these limitations, recent efforts have focused on integrating radiomics with genomic data—a concept known as radiogenomics—to develop noninvasive surrogates for tumor genotyping [7, 12, 13, 14]. Most radiogenomic studies to date have relied on molecular data derived from tissue biopsy samples, considered the standard reference for genomic validation [15]. However, tissue sampling captures only a static and localized snapshot of tumor biology, which may not accurately reflect clonal heterogeneity or temporal evolution. In contrast, liquid biopsy—particularly through the analysis of circulating tumor DNA (ctDNA)—provides a dynamic and minimally invasive means of capturing tumor‐derived genetic material from the bloodstream [16, 17, 18, 19]. This approach has shown promise in identifying actionable mutations, monitoring treatment response, and detecting resistance mechanisms, making it a valuable complement to imaging in NSCLC management. Despite the growing availability of both radiomic and ctDNA‐based data, their combined use in a radiogenomic framework remains underexplored. In particular, there is limited evidence on whether radiomic features extracted from standard‐of‐care CT scans can reflect genomic alterations identified through liquid biopsy [20, 21, 22, 23]. This represents a critical knowledge gap, especially considering that ctDNA may provide a more comprehensive representation of the tumor's molecular landscape than single‐site biopsies [24, 25, 26, 27]. In this exploratory study, we investigate the association between CT‐derived radiomic features and gene mutations identified through liquid biopsy in a cohort of treatment‐naïve NSCLC patients. Specifically, we aim to evaluate the feasibility of using radiomics as a noninvasive surrogate for ctDNA‐detected mutational status. We also examine the relative performance of radiomic models in classifying individual versus paired gene mutations, and analyze which classes of radiomic features—such as first‐order statistics and second‐order texture matrices—are most strongly implicated in these associations. By focusing on liquid biopsy as the molecular reference, our work offers a novel perspective within the field of radiogenomics, with potential implications for noninvasive tumor characterization and patient stratification. Furthermore, given the recognized complexity of oncogenic pathways and the increasing awareness of tumor heterogeneity, we extended our analysis to include combinations of gene mutations. This choice was based on the hypothesis that radiomic phenotypes may not only reflect individual driver mutations but also capture integrated biological signals shaped by co‐existing genetic alterations and the tumor microenvironment.

## 2 Methods

### 2.1 Patient Selection

The Ethical Review Board at the “Campania University Luigi Vanvitelli,” through internal resolution No. 24997/2020 dated 12.11.2020, granted approval for the commencement of this prospective study. In adherence to the sanctioned protocol, individuals diagnosed with histologically confirmed lung cancer, who had undergone both tissue CT‐guided biopsy and liquid biopsy, were enlisted for participation. The entire study adhered rigorously to pertinent guidelines and regulations.

The eligibility of potential participants was meticulously evaluated by the investigators. Inclusion criteria encompassed: (a) individuals aged ≥ 18 years, (b) possessing sound mental health, (c) having the capacity to provide informed consent through a specific form, (d) confirmed diagnosis of NSCLC through histological means, (e) undergoing computed tomography staging, and (f) willingness to provide a blood sample for liquid biopsy. Conversely, exclusion criteria comprised: (a) individuals below 18 years of age, (b) pregnant individuals, (c) those with an absolute contraindication to CT studies (due to prior adverse reactions to contrast medium), (d) individuals incapable of signing the specified informed consent form, (e) those declining to provide a blood sample for liquid biopsy, and (f) individuals lacking a conventional biopsy.

The research involved individuals newly diagnosed with a pulmonary nodule, scheduled for transthoracic CT‐guided Fine‐Needle Aspiration Cytology (FNAC) to confirm NSCLC. Detailed information regarding procedure risks, potential complications, and the likelihood of FNAC inadequacy was provided to the patients, who then provided written informed consent for the procedure.

A 18 Gauge semiautomatic tru‐cut needle was utilized consistently for all cases.

Subsequently, a radiologist, in collaboration with a pathologist, conducted CT‐guided percutaneous transthoracic FNAC procedures using a Revolution Discovery 64‐slice CT scanner (General Electric, Boston, MA, USA). ROSE (Rapid On‐Site Evaluation) was consistently conducted to determine the number of passes and vial selection during CT‐guided cases. As per our established protocol [28], each patient underwent 1–4 passes. For ROSE, 1–2 smears were air‐dried and DiffQuik‐stained, while additional smears were alcohol‐fixed and Papanicolaou‐stained. Material from the initial pass hub and subsequent passes was preserved in formalin for cell block (CB) preparation.

Following the procedure, patients were monitored in the recovery room, and a chest radiography was performed 2 h later to detect any potential complications.

In our prospective study, 60 patients were recruited, including 44 with lung adenocarcinoma, 14 with squamous cell carcinoma, and two with adenosquamous carcinoma. Among them, 16 were at Stage I, 11 at Stage II, 16 at Stage III, and 17 at Stage IV. Tumor localization revealed that 47 had peripheral tumors, while 13 had central tumors. Of the patients, 30 were former smokers, 27 were current smokers, and five were nonsmokers. The average age of the patients was 68 years.

### 2.2 Blood Samples and ctDNA Extraction

For blood sample collection, 5 mL of blood was drawn using ethylenediaminetetraacetic acid (EDTA) blood collection tubes on the same day as the CT‐guided Fine‐Needle Aspiration Cytology procedure.

The blood underwent centrifugation at 1800*g* for 10 min at 4°C to eliminate blood cells. Subsequently, the supernatant underwent further centrifugation at 16,000*g* for 10 min at 4°C to ensure the removal of any residual cells. The extraction of ctDNA involved digesting 2 mL of plasma in 100 μL of proteinase K buffer for 10 min at 37°C, followed by purification utilizing the Plasma XS kit as per the recommended protocol. The quantification of the purified ctDNA was carried out using a Picogreen fluorescence assay with lambda DNA standards provided by the kit.

### 2.3 ctDNA Sequencing and Analysis

The 5‐biotinylated probe solution contained capture probes targeting cancer‐related genes. The bait sequences aimed at specific genetic regions. Hybridization, target amplification, barcode library preparation, and size selection were conducted following the manufacturer's instructions. Utilizing the Tru Sight Oncology 500 ct DNA kit, which focuses on target enrichment encompassing 523 cancer‐related genes, libraries were prepared. The sequencing assay aimed to identify various genetic mutations, including small nucleotide variants (SNVs), indels, splice variants, and key biomarkers such as tumor mutational burden (TMB) and microsatellite instability (MSI). The sequencing process was performed using an Illumina Nova Seq 6000 platform from San Diego, CA, USA. The subsequent analysis utilized the Tru Sight Oncology local app on a Dragen server.

### 2.4 Image Acquisition

Imaging was conducted using a Revolution HD CT scanner (GE Medical Systems) at our institution, adhering to a standardized protocol. The equipment was set to operate in helical mode with a tube voltage of 120 kVp and a pixel spacing range of 0.578–0.976 mm. Each patient underwent a high‐resolution chest CT without contrast in the supine position to evaluate the location, size, and number of lesions. The protocol included thin‐section imaging with a slice thickness of 1.5 mm, a rotation time of 200–500 ms, a matrix size of 512 × 512, collimation of 1.5–3 mm, and a field of view (FOV) of 35 cm. A high spatial frequency reconstruction algorithm was applied, and all scans were acquired during full inspiration to ensure consistency.

### 2.5 Image Segmentation

**FIGURE 1:**
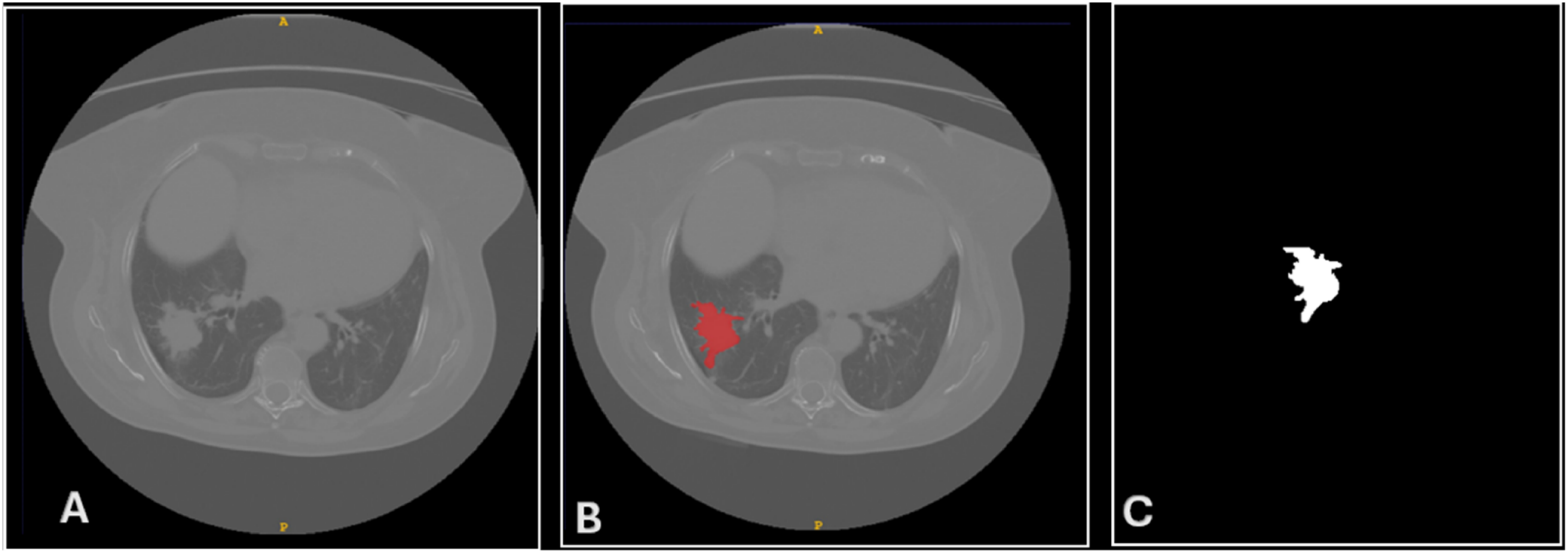
(A) Axial CT image revealing a lesion with irregular margins highly suspicious for lung cancer, subsequently confirmed as adenocarcinoma upon histological examination. (B) Manual segmentation of the lesion performed using itk‐snap segmentation software, highlighting its precise delineation. (C) Volumetric rendering of the segmented lung tumor, providing a comprehensive visual representation of its spatial extent.

A skilled radiologist with 20 years of experience manually outlined lung lesions using the ITK‐SNAP package (obtainable at http://www.itksnap.org/) [29]. For segmentation purposes, only one lesion was chosen from each patient's volume, and the segmentation of Volumes of Interest (VOIs) encompassed all lesion slices (Figure 1). Following lesion delineation, radiomic features were extracted. The package Pyradiomics (obtainable at https://pyradiomics.readthedocs.io/) has been used [30]. Pyradiomics is compliant with the IBSI guidelines for texture feature calculation. These radiomic features were derived from original images [31]. No reinterpolation has been applied. In addition to first‐order histogram‐based features, texture features encompassed various matrices such as the gray‐level co‐occurrence matrix (GLCM), gray‐level size zone matrix (GLSZM), gray‐level run‐length matrix (GLRLM), neighboring gray‐tone difference matrix (NGTDM), and gray‐level dependence matrix (GLDM). A total of 107 features have been calculated for each lesion. According to standard analysis procedures, they were scaled, a transformation was applied to address asymmetries in the distribution, and highly correlated features in the subset sample were excluded (see Section 2.6).

### 2.6 Image Preprocessing

Prior to feature extraction, all CT images were resampled to isotropic voxel spacing (1 × 1 × 1 mm^3^). Intensity values were normalized using z‐score normalization, and a fixed bin width of 25 Hounsfield Units (HU) was applied for discretization.

### 2.7 Radiomic Features Extraction

A total of 107 radiomic features were extracted from each segmented lesion using PyRadiomics, following IBSI‐compliant guidelines. The features included first‐order statistics, shape, and texture features (GLCM, GLRLM, GLSZM, GLDM, and NGTDM). To ensure compliance with IBSI standards and improve reproducibility, all CT images were resampled to a uniform isotropic voxel size of 1 × 1 × 1 mm^3^ using linear interpolation. Prior to feature extraction, image intensities were normalized using z‐score normalization. Gray level discretization was performed using a fixed bin width of 25 HU. No additional filtering (e.g., wavelet or LoG filters) was applied to the images prior to radiomic analysis. All preprocessing and feature extraction steps were performed using PyRadiomics default settings unless otherwise specified.

Radiomic features were extracted using PyRadiomics (version 3.0.1), adhering to the standards of the Image Biomarker Standardization Initiative (IBSI). A total of 107 features were extracted per patient, encompassing:

- First‐order statistics (e.g., entropy, mean, skewness),
- Shape‐based features (e.g., surface area, sphericity),
- Second‐order texture features derived from GLCM, GLRLM, GLSZM, GLDM, and NGTDM matrices. Features were calculated in 3D using isotropic spacing.

### 2.8 Segmentation Repeatability Assessment

To evaluate intrareader segmentation repeatability, a subset of 20 randomly selected cases was re‐segmented by the same radiologist after a minimum wash‐out period of 2 weeks. The Dice Similarity Coefficient (DSC) was computed to assess spatial overlap between original and repeated segmentations.

### 2.9 Feature Selection and Classification

To reduce dimensionality, Least Absolute Shrinkage and Selection Operator (LASSO) regression was applied after z‐score standardization of all features. Classification models were constructed to predict both individual gene mutations and gene‐pair combinations (e.g., EGFR–ROS1, ALK–KRAS). Three machine learning classifiers were evaluated: support vector machine (SVM), linear discriminant analysis (LDA), and k‐nearest neighbors (kNN). Model performance was assessed using repeated 5‐fold stratified cross‐validation (10 repetitions). Evaluation metrics included accuracy, area under the receiver operating characteristic curve (AUC), sensitivity, and specificity.

In addition to single‐gene classification, we explored gene‐pair combinations to investigate whether radiomic features could capture more complex genomic signatures. Gene pairs were included only when all classes (both mutated, one mutated, none mutated) had at least 10 patients. This exploratory approach was motivated by the biological plausibility that certain imaging phenotypes may result from interactions between multiple mutations rather than single alterations.

### 2.10 Performance Evaluation Metrics

To provide a comprehensive evaluation of model performance beyond simple accuracy, we computed several classification metrics, including *ROC AUC, balanced accuracy, precision, recall*, and *confusion matrices* for each gene‐specific classification task. These metrics were derived from the cross‐validated predictions, and are reported for the best‐performing classifier per target gene.

### 2.11 Repoducibility and Availability

All analyses were conducted using Python (v3.9.6) and scikit‐learn (v1.0.2). Due to institutional privacy policies, raw imaging and genomic data cannot be shared publicly. However, anonymized datasets and analysis scripts are available upon reasonable request to the corresponding author.

This methodological workflow was designed to align with the CLEAR checklist for radiomic studies, promoting transparency and reproducibility (Table 1).

**TABLE 1:** CLEAR checklist for radiomic studies.

Section	CLEAR item	This study
1. Study design	Type of study and rationale	Retrospective, single‐center design; exploratory radiogenomic correlation study in NSCLC
2. Patient selection	Inclusion/exclusion criteria, flow of participants	60 patients with NSCLC enrolled between January 2020–June 2022; inclusion/exclusion clearly stated
3. Imaging protocol	Detailed scanner, acquisition parameters	Revolution Discovery 64‐slice CT (GE); 120 kVp; auto mAs; 1 mm slice thickness; contrast‐enhanced phase
4. Image preprocessing	Resampling, normalization, discretization	Resampled to 1 × 1 × 1 mm^3^; z‐score normalization; fixed bin width = 25 HU
5. Segmentation	Method, reproducibility, software	Manual segmentation by two expert radiologists using ITK‐SNAP; consensus on discrepancies; no reproducibility metric
6. Feature extraction	Software used, number/types of features, IBSI compliance	PyRadiomics v3.0.1; 107 features (first‐order, shape, texture); IBSI‐compliant
7. Feature selection	Dimensionality reduction techniques	LASSO regression applied after feature standardization
8. Modeling approach	Type of models used, training approach	SVM, LDA, kNN classifiers; stratified 5‐fold cross‐validation repeated 10 times
9. Performance evaluation	Metrics used and validation	Accuracy, AUC, sensitivity, specificity; internal validation only
10. Validation strategy	Internal/external validation, generalizability	Internal validation only; external validation noted as a future direction
11. Reproducibility	Code/data availability	Code and data not publicly available due to privacy policies; accessible upon request
12. Ethical approval	Ethical compliance, consent	Approved by Ethical Review Board at Campania University Luigi Vanvitelli (No. 24997/2020, 12.11.2020); consent waived

### 2.12 Statistical Analysis and Machine Learning

First, we selected gene‐mutations having an appropriate balance (adequate for subsequent steps in machine learning) between mutated/non mutated patients. Starting from 57 patients, we considered mutation having at least 20 patients in both classes. Among the 45 genes available, the following six had appropriate balance (see Section 3).

**FIGURE 2:**
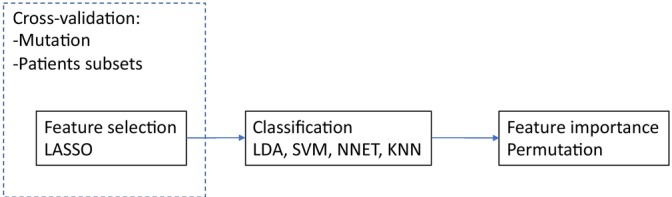
Pipeline followed in machine learning analysis. Particular care has been put in radiomic feature selection via the LASSO algorithm. For improved robustness of results we enclosed the feature selection into cross‐validation based on repeated resampling of patient subset and using different gene mutations as target. In the subsequent classification step we examined several among the most spread classifiers (LDA, SVM, Neural Networks, kNN, and Random Forest, using each gene mutation as target). For the best performing combination gene‐classifier we analyzed feature importance using a target shuffling approach. See text for more details.

To capture the association between radiomic features and gene mutations, we employed the pipeline illustrated in Figure 2. The pipeline begins with radiomic feature extraction from imaging data, where detailed quantitative features are derived to characterize tumor properties. Following feature extraction, a robust feature selection process using the LASSO algorithm is applied. This step ensures the identification of the most relevant features while minimizing redundancy and noise. To enhance reliability, the LASSO‐based selection is incorporated into a repeated 5‐fold cross‐validation framework, ensuring that the selected features remain consistent across different patient subsets and mutation targets.

The selected features are then analyzed in association with various gene mutations. This multitarget approach allows for the identification of potential relationships between radiomic features and genetic alterations, emphasizing the heterogeneity of lung cancer and its implications for patient‐specific therapeutic strategies.

LASSO is a robust shrinkage method that assigns zero weight to nonessential features, effectively isolating the most relevant predictors. Its use is motivated by its ability to handle noisy, high‐dimensional data efficiently and its proven effectiveness in radiomics and medical imaging, as demonstrated in the literature. For improved robustness and to ensure independence from the specific gene mutation chosen as the target, we embedded the LASSO feature selection within a cross‐validation framework based on repeated 5‐fold resampling of patient subsets, using various gene mutations as targets.

In this framework, for each combination of target mutation and cross‐validation fold, a unique subset of features was selected. The union of all these subsets was then used in subsequent steps. This approach leverages LASSO's robustness and sparseness while addressing the challenges posed by high‐dimensional radiomic data, ensuring that the selected features remain relevant and stable across varying conditions.

In the classification step we examined several among the most spread and well‐known classifiers (LDA, SVM, Neural Networks, kNN) using selected gene mutations as target. All processing has been written in R [32] repeated 5‐fold cross‐validation training of classifiers has been performed using the package CARET [33]. For the best performing combinations gene/classifier we analyzed feature importance using a target shuffling approach [34]. Feature importance corresponds to a specific model (classifier and feature) obtained shuffling each feature in turn and registering performance variation: shuffling of important features produce large variation of performance. Feature importance has been evaluated using the R package IML [35]. The above described analysis has been applied also to gene‐pairs with the aim to possibly catch eventual association between gene‐interaction and radiomics.

## 3 Results

### 3.1 Patient Characteristics

A total of 66 patients were initially screened according to the study criteria. After excluding six due to diagnostic ambiguities or neuroendocrine differentiation, two for technical issues in genetic analysis, and one for loss to follow‐up, 57 patients with confirmed NSCLC were included in the final analysis. A comprehensive overview of clinical and pathological characteristics is presented in Table 2.

**TABLE 2:** Clinico‐pathological characteristics of the analyzed series.

Characteristic	No.	%
Age		
≤ 70	31	54.4
≥ 70	26	45.6
Gender		
Male	39	68.4
Female	18	31.6
Histotype		
Adenocarcinoma	42	73.7
Squamous	15	26.3
PS ECOG		
0	32	56.1
1	18	31.6
2	7	12.3
Smoking		
Nonsmoker	5	8.8
Former smoker	27	47.4
Current smoker	25	43.8
Stage		
I/II	22	38.6
III	18	31.6
IV	17	29.8
*T*		
1/2	33	57.9
3	8	14.0
4	11	19.3
Nondefinable	5	8.8
*N*		
0	32	56.1
1	4	7.0
2	14	24.6
Nondefinable	7	12.3
EGFR mutation status		
Mutated	4	7.1
Wild‐type	52	92.9
Type of front‐line treatment		
Surgery	26	45.6
Platinum‐based CT	10	17.5
Active palliative treatment	4	7.0
CT and/or RT followed by surgery	4	7.0
Target therapy	3	5.3
ICI monotherapy	3	5.3
Nonplatinum‐based CT	2	3.5
Surgery followed by CT and/or RT	3	5.3

*Note:* Clinical and/or pathological *T* and *N* were not clearly definable in some cases because of tumor location, interobserver discordance, and ambiguities in clinical data leading to a final unresolved uncertainty.

Among the enrolled patients, 54.4% were younger than 70 years, and 45.6% were 70 years or older. The majority were male (68.4%) and had a history of smoking (91.2%). Adenocarcinoma represented the predominant histology (73.7%), followed by squamous cell carcinoma (26.3%). Performance status (ECOG) was 0 in 56.1%, 1 in 31.6%, and 2 in 12.3% of patients. Regarding disease stage, 38.6% were in stage I/II, 31.6% in stage III, and 29.8% in stage IV. T classification was pT1/2 in 57.9%, pT3 in 14.0%, and pT4 in 19.3%, while pN classification showed 56.1% pN0, 24.6% pN2, and smaller subsets in other categories. EGFR mutations were identified in four patients. Treatment decisions varied based on staging and mutation status, ranging from definitive local approaches to systemic therapy.

### 3.2 Radiomic Feature Extraction and Model Development

**FIGURE 3:**
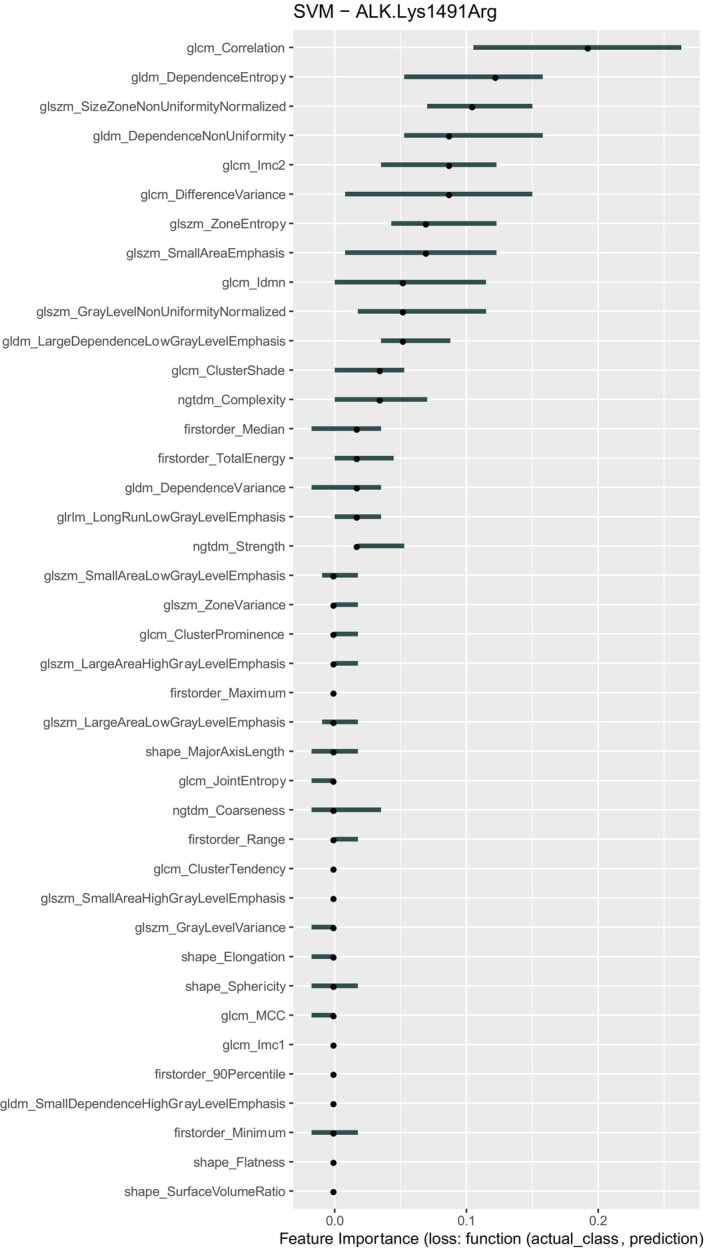
Feature importance analysis for the best performing classifier—target (LDA and ALK Lys) in Table 2. Feature importance is obtained shuffling each feature in turn and registering performance variation: Shuffling of important features produce large variation of performance. See text for details.

**FIGURE 4:**
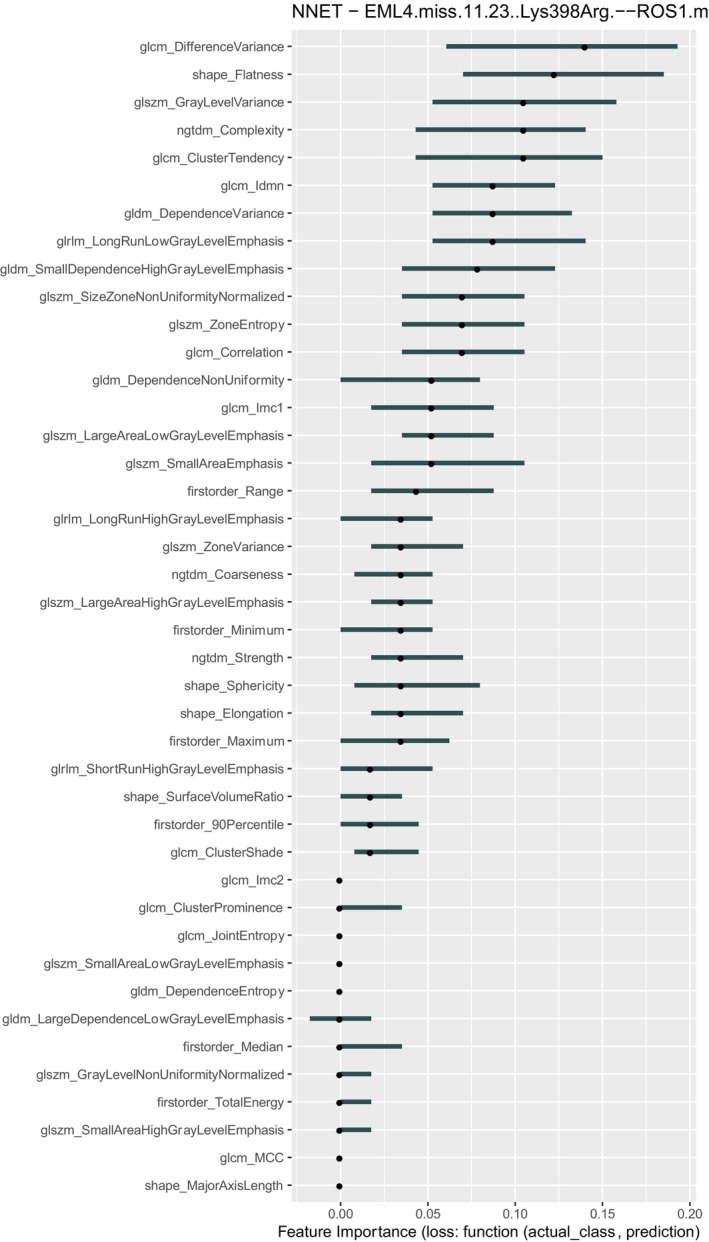
Feature importance analysis for the best performing classifier—target (NNET and EML4‐ROS1Lys) in Table 2. Feature importance is obtained shuffling each feature in turn and registering performance variation: Shuffling of important features produce large variation of performance. See text for details.

For each of the 57 patients, 107 radiomic features were extracted from contrast‐enhanced CT images. Only genes with sufficient class balance (i.e., ≥ 20 patients in both mutated and wild‐type groups) were retained for analysis. Among 45 initially available genes, six passed this threshold: EGFR, ALK, EML4, and ROS1 (with some overlap and redundancy due to fusion variants, see Table 3). Using a robust pipeline (illustrated in Figure 2), we applied 50‐times repeated 5‐fold cross‐validation with LASSO feature selection. A total of 40 features were selected for the classification task using single‐gene mutation as the target. Three classifiers were trained (SVM, LDA, and kNN), and performance metrics were evaluated. Overall, model performance was modest across all genes, with classification accuracies generally below 0.65. The best result was observed using LDA for the ALK‐Lyn mutation class, reaching an accuracy of approximately 0.60 (Figures 3 and 4).

**TABLE 3:** Accuracy of the classifiers with respect to single‐gene mutation. Heatmap displaying the significance of the association between gene mutations and radiomic features for each classifier. Higher values (closer to 0.60) indicate stronger associations, while lower values (closer to 0.42) suggest weaker associations.

	EGFR	ALKasp	ALKlys	EML4	ROS1Lys	ROS1Ser
LDA	0.42	0.46	0.49	0.52	0.45	0.44
SVM	0.44	0.52	0.60	0.44	0.45	0.44
NNET	0.47	0.58	0.59	0.48	0.49	0.49
kNN	0.46	0.59	0.60	0.51	0.50	0.56
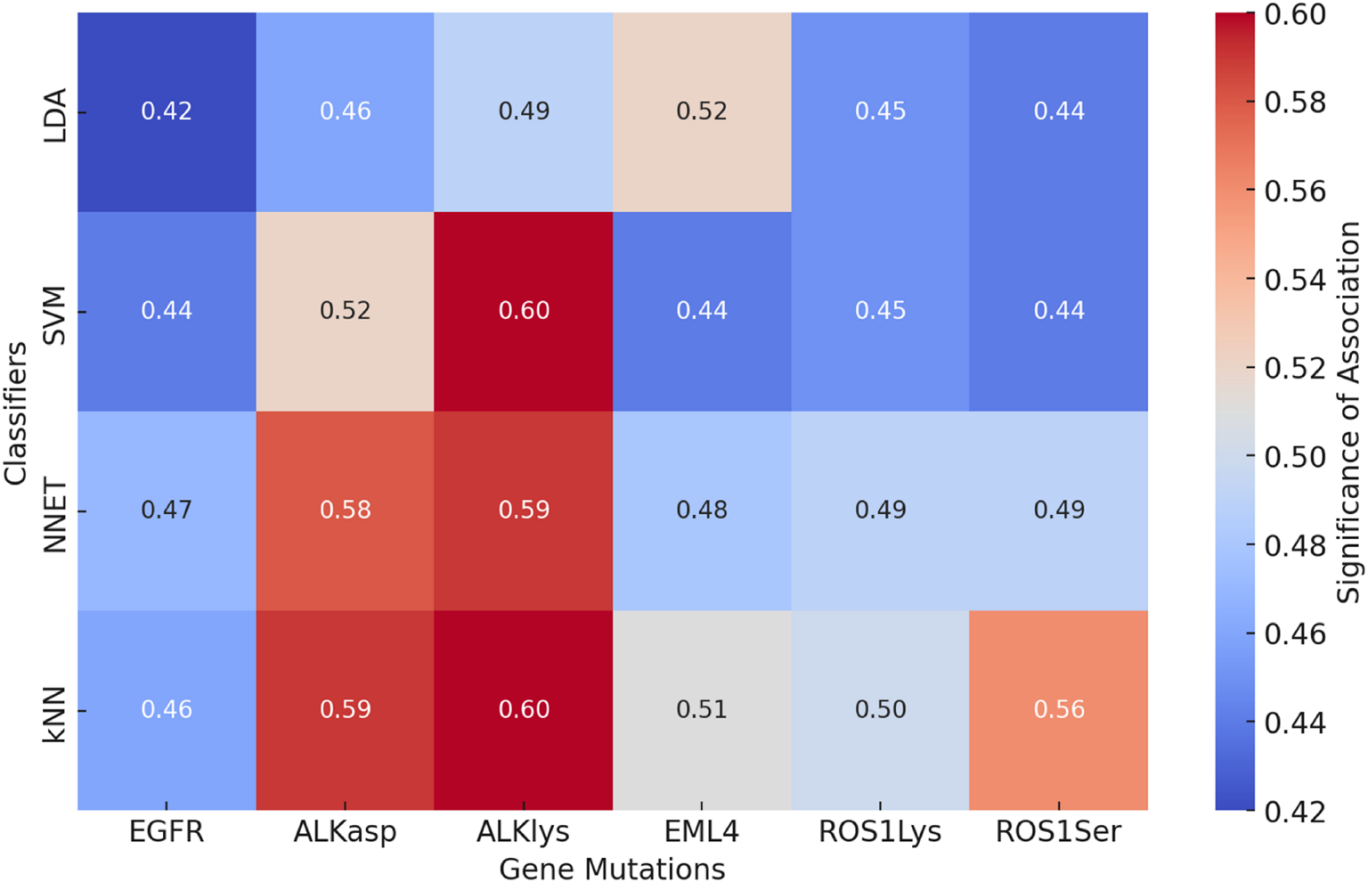						

### 3.3 Segmentation Repeatability Results

Intrareader repeatability was evaluated on 20 randomly selected cases. The average DSC between original and re‐segmented tumor volumes was 0.89 ± 0.03, indicating excellent agreement and high spatial consistency in manual segmentation.

### 3.4 Feature Importance Analysis

**FIGURE 5:**
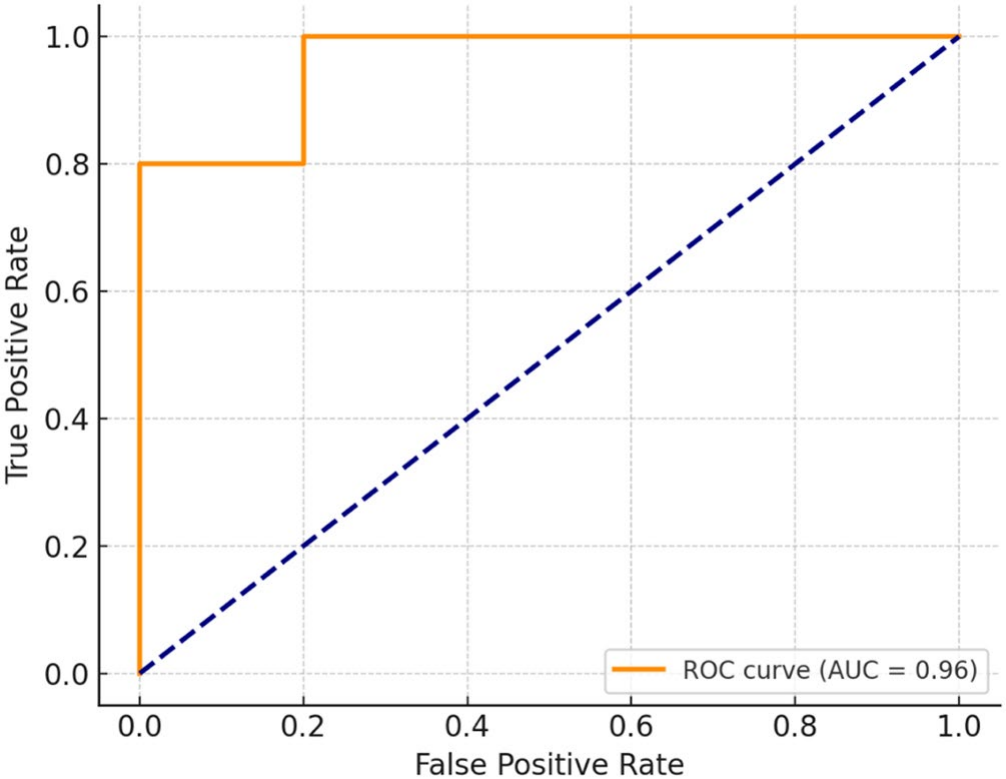
ROC curve showing the discriminative performance for ALK mutation (AUC = 0.96).

Feature importance for the best‐performing model (LDA with ALK‐Lyn) is shown in Figure 5. The most predictive features were predominantly second‐order texture metrics, including glcm‐correlation, gldm‐dependenceEntropy, and glszm‐SizeZoneNonUniformityNormalized. This suggests that specific textural patterns on CT imaging may reflect underlying genomic alterations, although the strength of association remains limited.

### 3.5 Gene‐Pair Classification Analysis

We extended the analysis to pairs of gene mutations, considering combinations with sufficient class balance (at least 10 patients per class across all combinations: both mutated, one mutated, neither mutated). Six pairs met inclusion criteria: EGFR–ALK, EGFR–EML4, EGFR–ROS1, EML4–ROS1 (some repeated due to overlapping transcript variants). In this context, 42 radiomic features were selected by LASSO. Classifier performance across all gene pairs was lower than in the single‐gene models. Accuracy did not exceed 0.50 in any case, indicating poor discriminative power of radiomic features when classifying dual‐gene mutation status (Table 4). Feature importance analysis again highlighted texture features, but without consistent patterns across pairs.

**TABLE 4:** Accuracy of the classifiers with respect to gene‐pair mutation. Heatmap displaying the significance of association between gene‐pair mutations and radiomic features for each classifier. Higher values indicate stronger associations.

	EGFR‐ALKasp	EGFR‐EML4	EGFR‐ROS1Lys	EGFR‐ROS1Ser	EML4‐ROS1Lys	EML4‐ROS1Ser
LDA	0.20	0.23	0.20	0.20	0.25	0.24
SVM	0.24	0.20	0.19	0.19	0.23	0.22
NNET	0.29	0.21	0.22	0.24	0.30	0.29
kNN	0.28	0.23	0.24	0.25	0.27	0.30
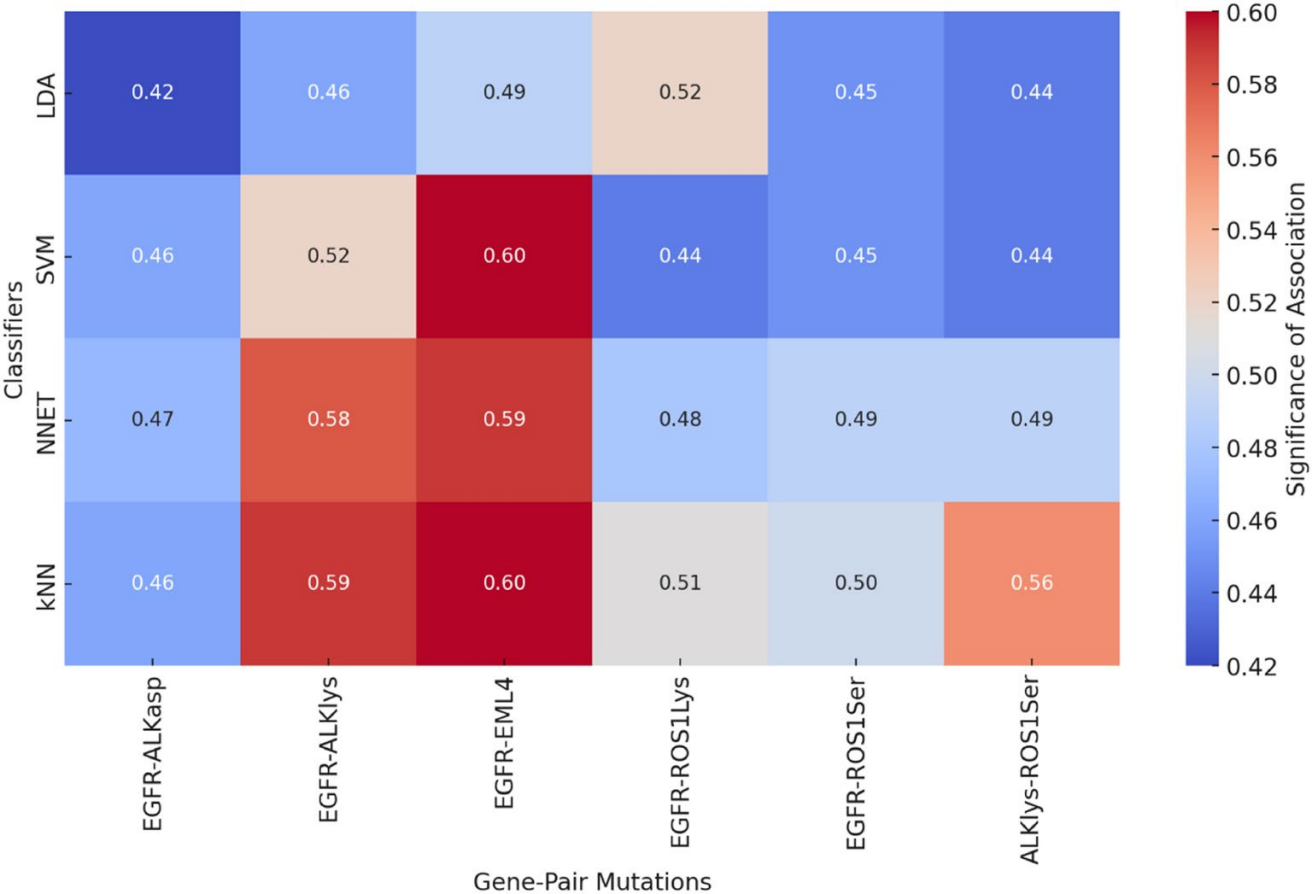						

### 3.6 Comprehensive Classification Metrics

To enhance the interpretability and robustness of the predictive analysis, additional performance metrics were calculated beyond classification accuracy. These included ROC AUC, balanced accuracy, precision, recall, and confusion matrices, reported for the best‐performing classifier—LDA applied to ALK‐Lyn mutation prediction.

The LDA model achieved a ROC AUC of 0.96, indicating excellent discriminatory capacity. The balanced accuracy was 0.90, with precision = 0.80 and recall = 1.00 for the mutated class. The confusion matrix shows high concordance between predicted and actual mutation status. The ROC curve (Figure 5) further confirms the model's sensitivity and specificity profile.

These comprehensive metrics provide a more nuanced assessment of model performance, particularly in the presence of potential class imbalance, and complement the earlier‐reported accuracy values.

## 4 Discussion

This study investigated the potential associations between radiomic features extracted from preoperative contrast‐enhanced CT images and genetic alterations detected through liquid biopsy in NSCLC patients. Our approach focused on both individual gene mutations and gene‐pair combinations, with the goal of identifying imaging phenotypes reflective of underlying molecular profiles. The study yielded moderate classification performance when using radiomics to predict single‐gene mutations, and limited accuracy for gene‐pair classification. These results clearly indicate that the associations uncovered are preliminary and should be interpreted as exploratory, without claiming clinical applicability at this stage.

The selection of specific gene pairs such as EGFR–ALK, EGFR–ROS1, and EML4–ROS1 was informed by their recognized role as oncogenic drivers in NSCLC and their clinical relevance in targeted therapy. While often considered mutually exclusive, co‐alterations or subclonal heterogeneity involving these genes have been reported in the literature [36, 37, 38, 39, 40, 41]. Exploring their combined radiomic signature—although exploratory—offers a window into the polygenic interactions that may shape tumor phenotype and progression. This strategy reflects a broader view of oncogenesis as a multifactorial and dynamic process, which single‐gene analyses may fail to capture [42, 43, 44].

The analysis of gene pairs, although exploratory, was a deliberate methodological choice aimed at addressing the biological complexity of lung cancer. Oncogenesis is not solely driven by isolated genetic events but results from a dynamic interplay of multiple alterations and microenvironmental influences [45]. Considering the well‐documented intra‐ and intertumor heterogeneity, we hypothesized that radiomic features could potentially capture more integrated patterns of molecular complexity when modeling gene‐pair combinations. Despite the low classification accuracy for gene pairs (~0.30), the approach aligns with current perspectives on polygenic oncogenic mechanisms and provides a basis for future hypothesis‐driven studies.

The feature importance analyses consistently highlighted the role of second‐order texture features (e.g., GLCM, GLRLM, GLSZM metrics) across both single‐gene and gene‐pair models. These findings reinforce the hypothesis that tumor texture heterogeneity—possibly influenced by genetic mutations—may be partially detectable through radiomic analysis. However, the predictive power of these features was not sufficient to support robust clinical applications at this stage.

The use of LASSO regression for feature selection was grounded in its robustness in handling high‐dimensional data, particularly in small datasets. By enforcing sparsity and controlling overfitting, LASSO enabled the selection of a stable subset of features, which is crucial in radiomic pipelines where collinearity and redundancy are common.

While our radiogenomic analysis relied exclusively on liquid biopsy data, we recognize the relevance of comparing radiomic associations derived from tissue biopsy versus liquid biopsy. Unfortunately, due to the absence of comprehensive tissue‐based NGS profiling in our cohort, such a comparison was not feasible. Nevertheless, liquid biopsy was chosen because of its capacity to capture tumor heterogeneity and temporal genomic dynamics, aspects often missed by single‐site tissue sampling.

This study builds upon our prior work on the reproducibility of radiomic features with respect to segmentation variability, reinforcing the feasibility of incorporating radiomics into integrative oncologic pipelines. In the present study, we also performed an intrareader reproducibility assessment. A subset of 20 randomly selected cases was re‐segmented by the same reader after a 2‐week washout period. The resulting DSC demonstrated high concordance (mean DSC = 0.89 ± 0.03), confirming the repeatability of the manual segmentation approach.

While these results are promising as proof‐of‐concept, further validation is necessary to assess their reproducibility and generalizability. Specifically, external validation on larger, multi‐institutional cohorts is essential before considering any potential translation into clinical practice. Future investigations should also explore the integration of radiomics with other omics modalities and clinical data to enhance the robustness and interpretability of predictive models.

In conclusion, this study should be viewed as an exploratory investigation into the complex interplay between imaging phenotypes and liquid biopsy‐derived genomics in NSCLC. It highlights both the potential and current limitations of radiogenomics, emphasizing the need for rigorous methodological validation before clinical implementation can be considered.

## 5 Limitations

This study has several limitations that should be considered when interpreting the results. First, potential confounding variables such as age and gender may contribute to variability in the observed associations, which could influence the robustness of the findings. Second, while we utilized cross‐validation and regularization techniques, such as LASSO, to reduce the risk of overfitting during feature selection, the relatively small sample size (57 patients) remains a concern. This limitation underscores the need for cautious generalization of the results. Third, all data were collected from a single institution, which limits the external validity of the findings. The lack of multi‐institutional validation restricts the ability to generalize the results to broader populations, emphasizing the need for future studies to incorporate diverse and larger patient cohorts to ensure reproducibility and applicability in varied clinical settings.

## Author Contributions

Conceptualization: Maria Paola Belfiore, Mario Sansone, Alfonso Reginelli. Methodology: Mario Sansone, Giovanni Ciani, Vittorio Patanè. Software: Mario Sansone, Giovanni Ciani. Validation: Giovanni Ciani, Vittorio Patanè. Formal Analysis: Giovanni Ciani, Mario Sansone. Investigation: Maria Paola Belfiore, Carlotta Genco, Vittorio Patanè. Resources: Roberta Grassi, Giovanni Savarese, Marco Montella, Riccardo Monti. Data curation: Marco Montella, Giovanni Savarese, Roberta Grassi. Writing – original draft preparation: Vittorio Patanè, Maria Paola Belfiore. Writing – review and Editing: All authors. Visualization: Giovanni Ciani, Vittorio Patanè. Supervision: Alfonso Reginelli, Roberta Grassi. Project Administration: Alfonso Reginelli. Funding acquisition: Not applicable.

## Ethics Statement

The Ethical Review Board at the “Campania University Luigi Vanvitelli,” through internal resolution No. 24997/2020 dated 12.11.2020, granted approval for the commencement of this prospective study.

## Consent

Written informed consent was obtained from patients prior to the enrolment to this study.

## Conflicts of Interest

The authors declare no conflicts of interest.

## Data Availability

The data supporting the findings of this study are available from the corresponding author upon reasonable request.
